# The β-*N*-Acetylhexosaminidase in the Synthesis of Bioactive Glycans: Protein and Reaction Engineering

**DOI:** 10.3390/molecules24030599

**Published:** 2019-02-08

**Authors:** Pavla Bojarová, Natalia Kulik, Michaela Hovorková, Kristýna Slámová, Helena Pelantová, Vladimír Křen

**Affiliations:** 1 Laboratory of Biotransformation, Institute of Microbiology, Czech Academy of Sciences, Vídeňská 1083, CZ-14220 Praha 4, Czech Republic; michaela.hovorkova@natur.cuni.cz (M.H.); slamova.kristyna@gmail.com (K.S.); kren@biomed.cas.cz (V.K.); 2 Center for Nanobiology and Structural Biology, Institute of Microbiology, Czech Academy of Sciences, Zámek 136, CZ-37333 Nové Hrady, Czech Republic; kulik@nh.cas.cz; 3 Laboratory of Molecular Structure Characterization, Institute of Microbiology, Czech Academy of Sciences, Vídeňská 1083, CZ-14220 Praha 4, Czech Republic; pelantova@biomed.cas.cz

**Keywords:** β-*N*-acetylhexosaminidase, galectin-3, molecular modeling, site-directed mutagenesis, solvent, substrate specificity, transglycosidase

## Abstract

*N*-Acetylhexosamine oligosaccharides terminated with GalNAc act as selective ligands of galectin-3, a biomedically important human lectin. Their synthesis can be accomplished by β-*N*-acetylhexosaminidases (EC 3.2.1.52). Advantageously, these enzymes tolerate the presence of functional groups in the substrate molecule, such as the thiourea linker useful for covalent conjugation of glycans to a multivalent carrier, affording glyconjugates. β-*N*-Acetylhexosaminidases exhibit activity towards both *N*-acetylglucosamine (GlcNAc) and *N*-acetylgalactosamine (GalNAc) moieties. A point mutation of active-site amino acid Tyr into other amino acid residues, especially Phe, His, and Asn, has previously been shown to strongly suppress the hydrolytic activity of β-*N*-acetylhexosaminidases, creating enzymatic synthetic engines. In the present work, we demonstrate that Tyr470 is an important mutation hotspot for altering the ratio of GlcNAcase/GalNAcase activity, resulting in mutant enzymes with varying affinity to GlcNAc/GalNAc substrates. The enzyme selectivity may additionally be manipulated by altering the reaction medium upon changing pH or adding selected organic co-solvents. As a result, we are able to fine-tune the β-*N*-acetylhexosaminidase affinity and selectivity, resulting in a high-yield production of the functionalized GalNAcβ4GlcNAc disaccharide, a selective ligand of galectin-3.

## 1. Introduction

*N*-Acetylhexosamine oligosaccharides are attractive synthetic targets since they act as potent ligands of biotechnologically or biomedically attractive lectins [1,2]. β-*N*-Acetylhexosaminidases (EC 3.2.1.52) have the advantage of tolerating a number of functional groups incorporated in both the donor and acceptor carbohydrate molecule [3,4,5]. Besides their natural hydrolytic activity, they can act in the synthetic (transglycosylation) mode, performing glycosylations of a range of acceptors [6,7]. Suitable modifications at glycan C-1 may be applied for covalent attachment of glycans to a multivalent carrier, resulting in multivalent glycomimetics with enhanced affinity to lectin targets [8,9].

We have recently discovered that a point mutation of the active-site amino acid Tyr to other amino acid residues, especially Phe, His, and Asn, can strongly suppress the hydrolytic activity of β-*N*-acetylhexosaminidases and favorably shift the reaction equilibrium towards the synthetic mode [10]. In this work, we have found that Tyr470 is also an important mutation hotspot for altering the intrinsic feature of β-*N*-acetylhexosaminidases—the ratio of activities towards *N*-acetylglucosamine (GlcNAc) and *N*-acetylgalactosamine (GalNAc) substrates. In most wild-type enzymes, this ratio is relatively undistinctive, ranging between ca 0.7–1.5 [11], though there are exceptions [12]. Moreover, the enzyme selectivity may further be manipulated by reaction engineering; the GalNAcase/GlcNAcase activity ratio is strongly dependent on pH and especially on the presence of organic co-solvents in the reaction medium. We demonstrate the utility of this approach on the β-*N*-acetylhexosaminidase from *Talaromyces flavus* (*Tf*Hex), recombinantly expressed in *Pichia pastoris* [13]. This enzyme has exhibited an exceptionally broad substrate specificity and synthetic utility, even as a wild type [14]. By combining the protein and reaction engineering approaches, we were able to prepare a functionalized GalNAcβ4GlcNAc disaccharide in an over-100-mg yield from a chemically functionalized acceptor in a single reaction step. 

GalNAc-terminated oligosaccharide epitopes rarely occur in mammalian glycoproteins [15,16] or parasites [17] and are classified as specific biomarkers of cancer [18]. The GalNAcβ4GlcNAc (LacdiNAc) disaccharide epitope is selectively recognized by a therapeutically relevant galectin-3 [19,20]. Selective enzymatic GalNAc-ylations are a challenging task within the family of carbohydrate-active enzymes. This reaction may be accomplished using mutant glycosyltransferases [21], which, however, often lack the robustness, high-yield production, and broad acceptor specificity of β-*N*-acetylhexosaminidases.

The present work demonstrates the critical importance of the Tyr470Phe mutation in *Tf*Hex—not only for its synthetic capability but especially for the exceptional stabilization of its GalNAcase activity against organic solvents during the synthetic procedure. These features greatly enhanced the yield of preparative GalNAcylations and enabled a large-scale preparation of the desired LacdiNAc disaccharide, which may further be applied for multivalent presentation in galectin-3-binding glycomimetics. The altered substrate specificity of *Tf*Hex upon mutating the Tyr470 residue to Phe, His, or Asn was thoroughly studied by molecular modeling and substrate docking. 

## 2. Results

### 2.1. Production and Purification of TfHex Mutants

The genes of three Tyr470 mutants of *Tf*Hex, namely Tyr470Phe, Tyr470His, and Tyr470Asn, were cloned in the pPICZαA vector as described previously [10], and transformed into the competent cells of *P. pastoris* KM71H. For the preparative enzyme production, the respective enzymes were secreted into the minimal cultivation medium upon induction by methanol. The mutant variants were purified by cation-exchange chromatography at pH 3.5 in a single purification step, which yielded the proteins in a high purity for further biochemical characterization (for analysis by SDS-PAGE see also Figure S7 in the Supplementary Materials). The production yields and the specific GlcNAcase and GalNAcase activities are given in Table 1 along with the wild type *Tf*Hex for comparison. The results clearly demonstrate the efficiency of the *Pichia* producer for this type of enzymes, the production yields of pure enzymes reaching up to 10–39 mg/100 mL cultivation media (corresponding to approximately 70–85% purification yield). The specific activities given in Table 1 show that the ratio of GalNAcase/GlcNAcase activities strongly depends on the particular point mutation introduced. Whereas the wild type enzyme features GalNAcase/GlcNAcase activity ratio of 1.2, this ratio slightly declines to 1.0 in the Tyr470Phe mutant and rapidly falls to 0.3 and 0.1 in the His and Asn mutants, respectively, with the latter being a quite selective GlcNAcase. Notably, the low specific hydrolytic activities of all mutant enzymes compared to the wild type are expectable due to the fact that these mutants have a strongly suppressed hydrolytic activity compared to transglycosylation activity, and so, in fact, they behave like transglycosidases [10].

### 2.2. Biochemical Characterization of TfHex Mutants

**Figure 1:**
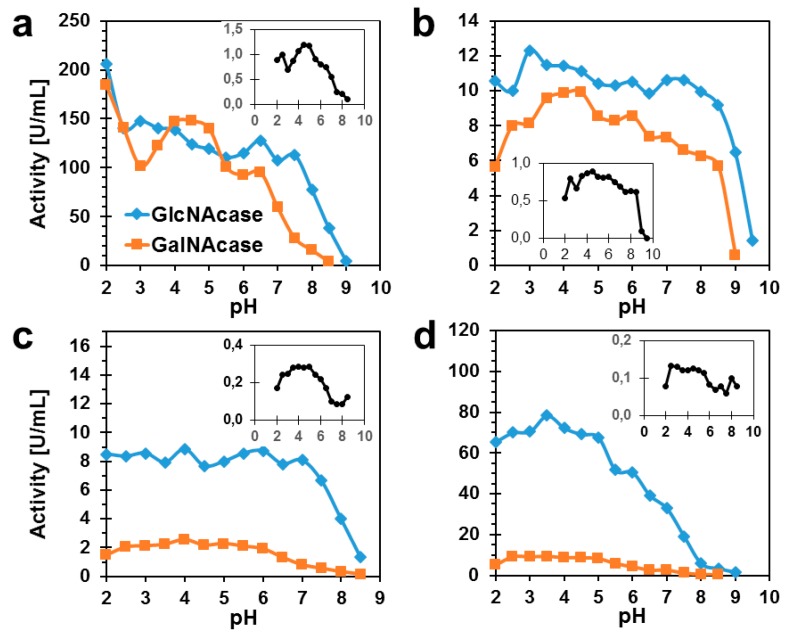
The profiles of pH optima of *Tf*Hex enzyme variants. (**a**) WT *Tf*Hex, (**b**) Tyr470Phe *Tf*Hex, (**c**) Tyr470His *Tf*Hex, (**d**) Tyr470Asn *Tf*Hex. The insets show the ratio of GalNAcase/GlcNAcase activities of the respective enzyme variant (Y-axis) in relation to pH (X-axis). All data were measured in triplicate.

The pH optima profiles of both GalNAcase and GlcNAcase activities of all the enzyme variants as well as of the wild type were determined as shown in Figure 1. These plots clearly show that the GalNAcase/GlcNAcase ratio, and, consequently, enzyme substrate selectivity, may be manipulated by altered pH. In all the *Tf*Hex variants tested, GalNAcase activity was at its peak at a slightly acidic pH (4–5) and it declined at pH approaching either lower or higher values. Thus, for example at pH 7, the ratio of GalNAcase/GlcNAcase declined approximately twice compared to pH 5 in the WT *Tf*Hex. At pH higher than 8, all enzyme variants were substantially inactivated. The by far highest GalNAcase activity at changing pH was found in the Tyr470Phe mutant. There, quite a stable ratio of GalNAcase/GlcNAcase activity was observed over a large span of pH 2.5–8.5. This finding clearly shows the importance of Tyr470Phe mutation for stabilizing and protecting the GalNAcase activity in *Tf*Hex. 

Furthermore, the kinetic parameters (*K*_m_, *k*_cat_) of the hydrolytic reactions with *p*NP-GlcNAc and *p*NP-GalNAc substrates with all enzyme variants were measured and compared to the wild type (Table 2).

Naturally, since the present mutant variants have a strongly suppressed hydrolytic activity, the hydrolytic catalytic efficiency is lower compared to the WT. It follows from the kinetic parameters that this behavior is caused mainly by reduction of *k*_cat_. In accordance with the activity ratios shown in Table 1, The GalNAcase activity was maintained best in Tyr470Phe *Tf*Hex, where the ratio of catalytic efficiencies towards *p*NP-GlcNAc and *p*NP-GalNAc practically copied the parameters of the WT (compare 434/150 to 12/3.3). This trend is also well visible on the values of *K*_m_. On the other hand, in both the His and Asn mutants, the GalNAcase activity is strongly suppressed compared to the GlcNAcase activity, which clearly reflects on the ratios of respective catalytic efficiencies (13/0.53 for Tyr470His, and 13/0.4 for Tyr470Asn *Tf*Hex). This considerable decline is caused by reduced affinity of the *p*NP-GalNAc substrate to the enzyme active site as reflected on the increased values of *K*_m_. These structure activity relationship conclusions were supported by in silico substrate docking and molecular modeling as shown further. 

### 2.3. Molecular Modeling and Docking of TfHex Mutants

For the modeling of the *Tf*Hex mutant variants and molecular dynamics simulations, the previously described homology model of *Tf*Hex [22] was used as a starting point. The calculated binding energies of substrates *p*NP-GlcNAc and *p*NP-GalNAc docked in the active site of WT and of mutant variants of *Tf*Hex are shown in Table 3. The binding scores of the respective hydrolytic products (GlcNAc or GalNAc) are included for comparison. There, a high binding energy implies an easy release of the hydrolytic product from the enzyme active site, and as a result, a faster catalytic turnover. The strongest binding was found for the complexes of WT-*p*NP-GlcNAc and Tyr470Phe-*p*NP-GalNAc. In contrast, the highest binding energies were observed for both substrates in the Tyr470Asn mutant, which agrees well with the higher *K*_m_ values (Table 2). Hence, we expect that the Tyr470Asn mutant accepts both substrates worse for hydrolysis than the WT and the other mutants. 

The binding affinity of the Tyr470His mutant to the substrates showed dependence on the protonation state of the histidine residue. The p*K*_a_ value of buried residues depends on the electrostatic potential formed by the surrounding residues [23]. The values predicted for p*K*_a_ of His470 by YASARA and PROPKA programs (incorporated in Schrödinger software) vary between 6.76 and 7.4. Though a better binding score was found for the neutral His form (Table 3), our calculations and all the experiments were performed at a slightly acidic pH, and hence we shall further assume a positively charged His residue. Notably, *p*NP-GalNAc exhibits a very weak interaction with the Tyr470His mutant, especially in the positive form (Table 3), which also reflects on the high *K*_m_ (Table 2). 

β-*N*-Acetylhexosaminidases adapt the conformation of substrates in the active site to allow hydrolysis and formation of specific interactions [24]. One of the important requirements is a close distance between the carboxylic oxygen of the catalytic residue (Glu371) and the substrate glycosidic oxygen [25]. Dynamics of this parameter for *Tf*Hex and the three mutant variants over a time span of 10 ns is depicted in Figure S8 (Supplementary Materials). It should be noted that the distance between the catalytic Glu371 and the substrate is not stable all the time and over longer molecular dynamics spans (over 7.5 ns) it tends to increase even for the WT. Nevertheless, the strongest interaction with the catalytic Glu371 was found in the complexes of WT-*p*NP-GlcNAc and Tyr470Phe-*p*NP-GalNAc, where *p*NP-GalNAc glycosidic oxygen needs longer to approach catalytic Glu371. The unfavorable orientation of *p*NP-GalNAc for hydrolysis by Tyr470His and Tyr470Asn mutants results in the increased distance of the glycosidic oxygen to catalytic Glu371 (>3.2 Å), and consequently, in the increase in *K*_m_ value. 

The flexibility of enzyme active site amino acids required to accommodate the substrate is characterized by the root-mean-square fluctuation of atomic positions (RMSF) of enzyme active-site residues (Figure S9, Supplementary Materials). In the complexes with the Tyr470Phe mutant, the optimum accommodation of substrates, especially of *p*NP-GalNAc, does not require a large reorientation of active site amino acids and features low RMSF values. Most of the studied residues are very stable over the equilibrated period of the molecular dynamics run (2–10 ns). The most flexible residues are Glu332 and Trp509, which are responsible for aglycon binding. The orientation of Trp509 is stabilized by a hydrogen bond network between its amide hydrogen, the C-6 hydroxyl of substrate, and the carboxylic oxygen of Asp472 [22]. The mutation of Tyr470 influences Asp472 orientation, and hence, Trp509 flexibility. A higher stability of Trp509 residue in Tyr470Phe *Tf*Hex probably results from the general stability of the active site in this mutant variant, especially when *p*NP-GalNAc is bound. Moreover, the presence of Phe may bring additional aromatic π-stacking with the Trp ring. In contrast, in the Tyr470Asn variant, Asp472 changes its orientation more significantly than in the other mutants and WT.

**Figure 2:**
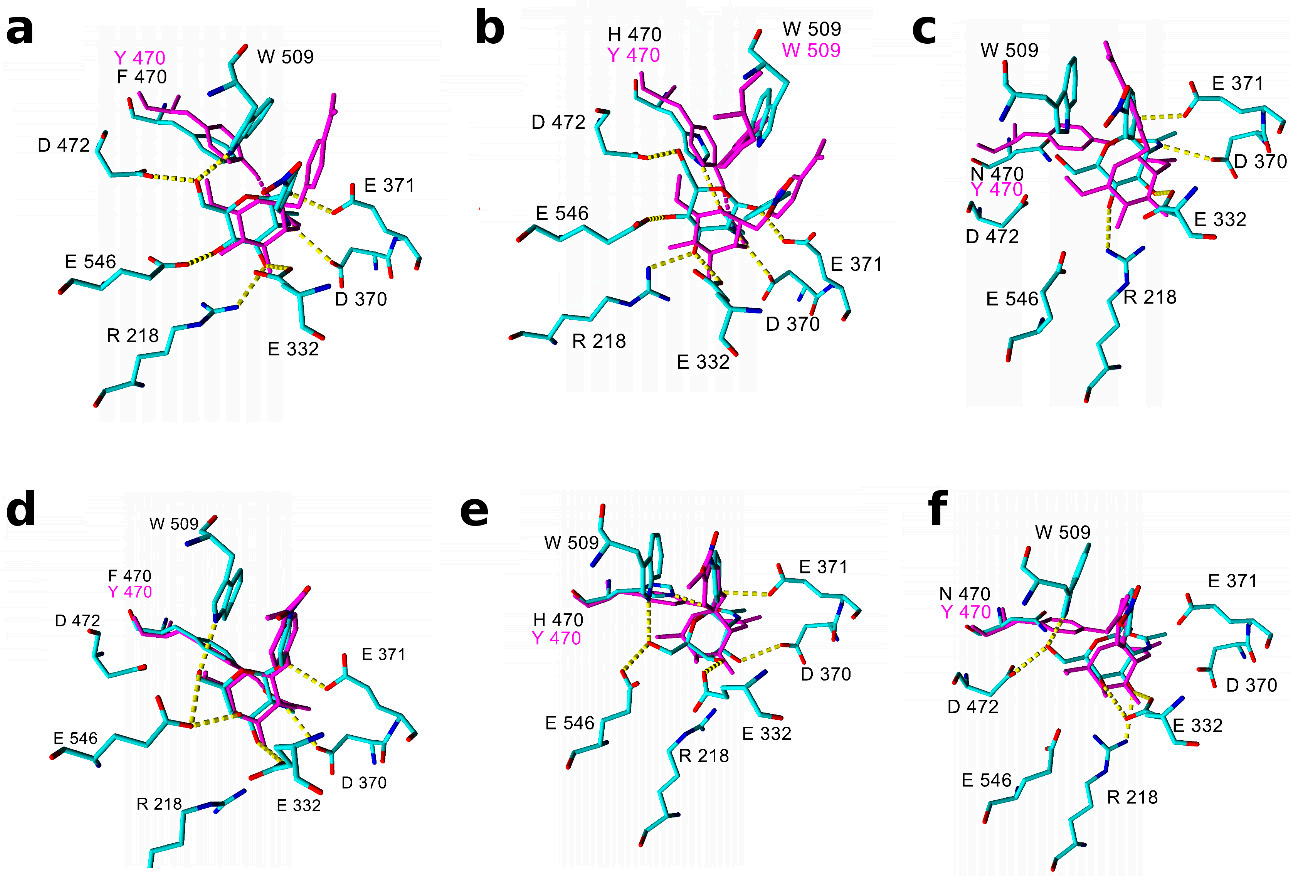
Complexes of *p*NP-GlcNAc and *p*NP-GalNAc in the active site of *Tf*Hex mutant variants from a stable period of molecular dynamics. *p*NP-GlcNAc in the active site of (**a**) Tyr470Phe mutant (**element colors**), of (**b**) Tyr470His mutant (positively charged; **element colors**), and of (**c**) Tyr470Asn mutant (**element colors**), all overlaid with the *p*NP-GlcNAc WT complex (**magenta**). *p*NP-GalNAc in the active site of (**d**) Tyr470Phe mutant (**element colors**), of (**e**) Tyr470His mutant (positively charged; **element colors**), and of (**f**) Tyr470Asn mutant (**element colors**), all overlaid with the *p*NP-GalNAc-WT complex (**magenta**). Amino acid residues of the WT enzyme are not shown if their orientation is similar to the mutant.

The substrates *p*NP-GlcNAc and *p*NP-GalNAc docked into the active site of mutant variants of *Tf*Hex are shown in Figure 2. For reference, the respective complexes with the WT are shown the Supplementary Materials (Figure S10). 

In the Tyr470Phe mutant, the orientation of substrates as well as the hydrogen bonding network are relatively similar to the WT (compare Figure 2a,d and Figure S10, Supplementary Materials). The mutation of Tyr470 to Phe hinders the formation of a hydrogen bond with the substrate *N*-acetyl group. The *p*-nitrophenyl group of *p*NP-GlcNAc is rotated with respect to Trp509 position in WT (Figure 2a) and the stacking with pyrrole of Trp509 is substituted by stacking with benzene after 7 ns (see also Figure S8). A more favorable binding score for *p*NP-GalNAc than for *p*NP-GlcNAc in the Tyr470Phe mutant along with the faster release of GalNAc hydrolytic product (demonstrated on its high binding energy, Table 3) explain the preference of *p*NP-GalNAc to *p*NP-GlcNAc by the Tyr470Phe mutant. 

In the complex with the Tyr470His mutant (Figure 2b,e), the *N*-acetyl group of both substrates is stabilized by hydrogen bonding with His470, which required reorientation of substrate in the active site with respect to WT. The incurred conformational changes of substrates and the reorganization of active site residues (especially of Trp509) led to the increased distance between the catalytic residue Glu371 and *p*NP-GalNAc (Figure S8). This distance is mostly too big for forming a stable interaction and decreases the probability of proton transfer from Glu371 to the substrate. Additionally, the GalNAc hydrolytic product binds quite strongly into the active site of the Tyr470His mutant (Table 3, Figure S11), which might imply its slow release from the active site, and consequently, a slower turnover of *p*NP-GalNAc substrate.

In the complex with the Tyr470Asn mutant (Figure 2c,f), water molecules are able to penetrate close to the substrate *N*-acetyl group and both substrates form a water-mediated hydrogen bond with Asn470. As a result, the substrates are shifted towards the *N*-acetyl binding place compared to the orientation in the WT and lose many hydrogen bonding interactions. *p*NP-GlcNAc is buried deeper in the active site than *p*NP-GalNAc in this mutant, which (together with a long distance between Glu371 and the glycosidic oxygen of *p*NP-GalNAc) may contribute to the mutant preference for *p*NP-GlcNAc substrate. 

### 2.4. Medium Engineering

**Figure 3:**
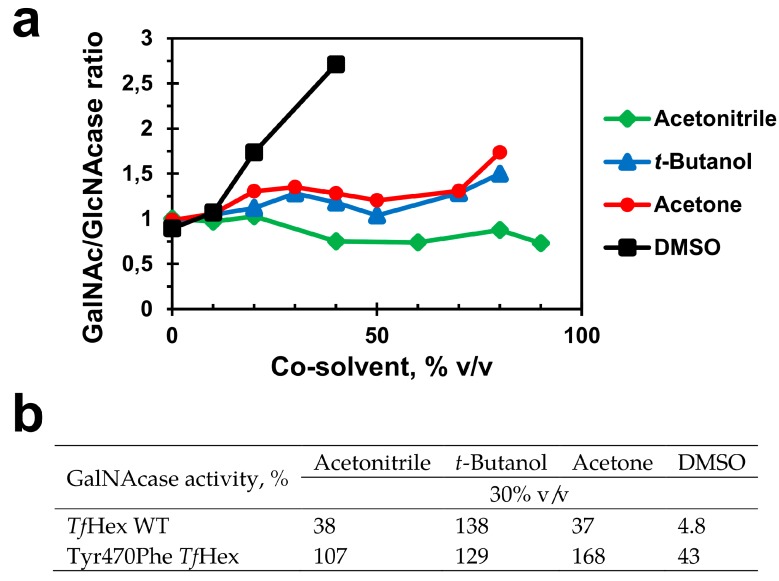
(**a**) Ratio of GalNAcase/GlcNAcase activity of Tyr470Phe *Tf*Hex in various concentrations of acetonitrile, acetone, *t*-butanol, and DMSO (0–80% *v*/*v*). (**b**) Residual GalNAcase activity (%) of WT *Tf*Hex Tyr470Phe *Tf*Hex in 30% *v/v* acetonitrile, acetone, *t*-butanol, and DMSO.

From the biochemical characterization as well as from the docking experiments, the Tyr470Phe mutant variant of *Tf*Hex was identified as the most promising candidate for a successful GalNAc-ylation reaction. Therefore, we decided to study the influence of various reaction media containing organic water-miscible aprotic co-solvents on the GalNAcase/GlcNAcase activity ratio of this mutant and compare it to the WT. Four solvents were selected for this aim: acetonitrile, acetone, *t*-butanol, and DMSO. The results are shown in Figure 3. 

The profiles of GalNAcase/GlcNAcase activity ratios at a varying concentration of co-solvents show a doubtless positive influence of the Tyr470Phe mutation on the stabilization of GalNAcase activity (Figure 2a). In all the cosolvent-buffer systems tested, the said ratio was maintained constant or even favoring the GalNAcase activity, such as in the case of DMSO (GalNAcase/GlcNAcase ratio of 2.7 at 40 % *v*/*v* DMSO). Astonishingly, the mutant enzyme kept a good activity even at high solvent concentrations (e.g., 99 % GalNAcase activity maintained in 80 % *v/v* acetone). This is quite a different case from what was observed in the WT *Tf*Hex (Figure 2b) and also in the other Tyr470 mutants tested (results not shown). Except for *t*-butanol, which as a protic alcohol solvent is not supposed to be very detrimental for enzyme activity, all the tested co-solvents considerably reduced GalNAcase activity in the WT *Tf*Hex at 30 % *v*/*v*. Notably, *t*-butanol is generally not very suitable as an inert co-solvent for transglycosylation reactions because, though sterically hindered, its enzymatic glycosylation has been described in the literature [26].

### 2.5. Enzymatic Synthesis of Functionalized Disaccharide ***3***

**Scheme 1:**
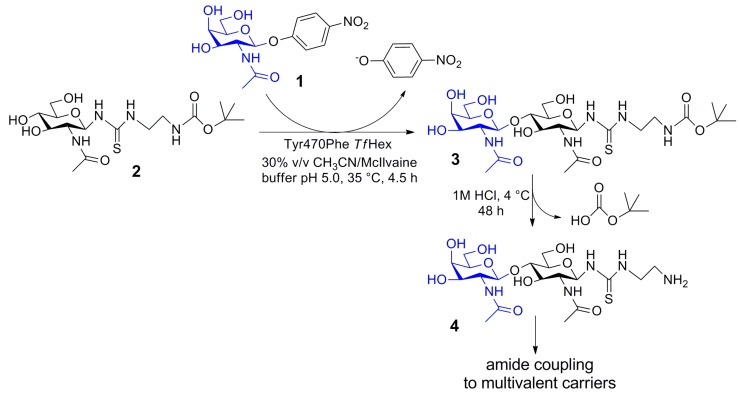
Upscaled synthesis of functionalized GalNAcβ4GlcNAc epitope (**3**) catalyzed by Tyr470Phe *Tf*Hex. Deprotection and coupling possibilities to biomaterials are implied.

The utility of the Tyr470Phe *Tf*Hex in the preparative GalNAc-ylation was demonstrated in a large-scale synthesis of a functionalized GalNAcβ4GlcNAc (LacdiNAc) epitope (**3**; Scheme 1). This disaccharide was recently found to act as a selective ligand of galectin-3, a therapeutically and diagnostically attractive lectin, in comparison to other galectins, in particular galectin-1 [21], and galectin-4 [27]. The reaction was run at pH 5 in the presence of 40 % *v*/*v* acetonitrile, which was ideal for maintaining GalNAcase activity and optimum for dissolving *p*NP-GalNAc substrate. For this particular reaction, higher concentrations of acetone as a co-solvent did not prove suitable due to evaporation problems, and in DMSO, surprisingly, the enzyme showed a much higher tendency of hydrolysis. Disaccharide **3**, which originated with 100 % regioselectivity in the reaction, was readily isolated in one step by gel permeation chromatography. Product **3** was afforded in an amount of 123 mg (51% isolated yield) and the reaction may further be scaled up. The thiourea linker may be deprotected under acidic conditions [19] and readily coupled to biomaterials [28] or other functionalized carriers to yield a multivalent glyco-nanomaterial selectively recognizing galectin-3, further applicable in biotechnology or biomedicine.

## 3. Discussion

It clearly follows from the present study that the impact of pH value, the presence of co-solvents, and other means of reaction engineering are strictly dependent on a particular enzyme-substrate combination and any general rules may hardly be applied. Thus, medium engineering must be performed with the particular substrate in question, and strictly speaking, no conclusions found for one substrate (e.g., *p*NP-GlcNAc) can be transferred to another substrate (e.g., *p*NP-GalNAc). Naturally, there are limits as to what results may be used for practical application in the upscaled synthetic reactions. For example, manipulation of reaction pH may not be practically applicable to a large extent, since many glycoside and oligosaccharides are relatively sensitive towards extreme pH values. Similarly, a considerable shift of the temperature of a synthetic reaction is hardly recommendable in the case of glycosidases. This is because higher temperatures generally lead to a lower transglycosylation yield in favor of hydrolysis, which is rather counterproductive when we aim at oligosaccharide synthesis. Nevertheless, a considerate choice of optimum reaction conditions may bring great benefits for reaction yields.

The upscaled synthesis of the functionalized LacdiNAc disaccharide presented herein is an outstanding example of an elegant, selective, and high-yielding enzymatic synthesis suitable for large-scale biotechnological application. Though the said product may also be prepared by a mutant Tyr284Leu β4-galactosyltransferase from human placenta [21], glycosyltransferases often face the issues of a low stability, mediocre production yields, and, in particular, a relatively high price of nucleotide phosphate donors. The synthesis presented herein has a comparable isolated yield and uses a much more priceworthy glycosyl donor of *p*NP-GalNAc-2 g for 300 USD for purchase at Carbosynth (Compton, Berkshire, UK), compared with UDP-GalNAc-10 mg for 315 USD or UDP-GlcNAc-250 mg for 415 USD. Notably, though less expensive, UDP-GlcNAc requires a tandem reaction with UDP-GlcNAc-4’-epimerase to convert to the desired UDP-GalNAc. After deprotection, the amino function may readily be used for coupling to biomaterials or surfaces to yield a defined multivalent ligand. Generally, glycosidases are not efficient in glycosylation of multivalent acceptors [29].

In this GalNAc-ylation reaction, the presence of organic co-solvent further lowered water activity in the reaction, besides manipulating the enzyme selectivity. Thus, the reaction equilibrium was shifted towards transglycosylation, and, what is more, the concentration of a badly water soluble *p*NP-GalNAc (maximum approximately 10 mM saturated water solution at 35 °C) was increased, which contributed to a high-yielding GalNAc-ylation. Glycosylation by fungal β-*N*-acetylhexosaminidases in the presence of organic co-solvents has often proved an advantageous strategy resulting in outstanding synthetic yields [30]. Importantly, the solvents employed as inert participants of glycosidase-catalyzed reactions preferably contain no hydroxyls—otherwise we face a risk of their glycosylation by the enzymes and formation of respective by-products. Even though the hydroxyl in *t*-butanol is strongly sterically hindered, and therefore its glycosylation is rather improbable, it was observed with fungal α-galactosidase [26]. Therefore, it is recommendable to opt for other co-solvents if possible. It appears that the combination of a Tyr-Phe mutation can not only considerably increase the synthetic yield of transglycosylation but also brings a vast stabilization of the enzyme under harsh reaction conditions, which makes it an ideal aid for constructing enzymatic synthetic tools. The exceptional stabilization is probably caused by the preservation of the stacking interaction with Trp509 of the hydrophobic environment in a Trp cluster of the active site pocket thanks to the phenyl moiety.

## 4. Materials and Methods 

### 4.1. Structural Characterization of Compounds

The electrospray mass spectrum was measured by the LTQ Orbitrap XL hybrid mass spectrometer (Thermo Fisher Scientific, Waltham, MA, USA). Methanol/water (4/1, *v*/*v*) was used as a mobile phase at a flow rate of 30 μL/min. The sample in methanol was injected into the mobile phase flow through a 2-μL loop under the following conditions: spray voltage 4.6 kV, capillary voltage −25 V, capillary temperature 275 °C. Tube lens voltage was −125 V for the negative ion mode or 150 V for the positive ion mode. The Xcalibur software (Thermo Fisher Scientific) was used for processing the data.

NMR analysis was performed on Bruker AVANCE III 400 and 700 MHz spectrometers (Bruker BioSpin, Rheinstetten, Germany) in D_2_O at 30 °C. Residual signals of D_2_O (*δ*_H_ 4.508 ppm) served as an internal standard; the carbon spectra in D_2_O were referenced to the acetone signal (*δ*_c_ 30.50). NMR data were analyzed by standard manufacturers’ software TopSpin 3.5. For improving resolution, two-parameter double-exponential Lorentz-Gauss function was applied for ^1^H. Line broadening (1 Hz) was applied to improve signal-to-noise ratio in ^13^C. The assignment of individual spin systems in the analyzed compounds was performed by COSY or 1d-TOCSY and transferred to carbons by HSQC. The assignment of quaternary carbons and interconnection of spin systems was done by HMBC experiment.

### 4.2. Production of TfHex Variants in Pichia pastoris

The yeast expression vectors pPICZαA (Invitrogen, Life Technologies, Carlsbad, CA, USA) carrying the genes of WT *Tf*Hex and its three mutant variants (Tyr470Phe, Tyr470His, Tyr470Asn) were prepared as described previously [10,13]. The constructs were linearized (15 µg) using restriction endonuclease *Sac*I (New England Biolabs, Ipswich, MA, USA), and electroporated into *P. pastoris* KM71H competent cells (Invitrogen, Life Technologies, Carlsbad, CA, USA) according to the manufacturer´s protocol (EasySelect Pichia Expression Kit; Invitrogen, Life Technologies, Carlsbad, CA, USA). The resulting colonies were tested for the enzyme production as described below. Two colonies with the highest production and purity of the respective enzymes as analyzed by SDS-PAGE and activity measurements were cryopreserved at −80 °C in 15 % (*v*/*v*) glycerol.

Recombinant *Tf*Hex variants were produced essentially according to the manufacturer´s instructions (EasySelect Pichia Expression Kit; Invitrogen, Life Technologies) [13]. For a small-scale production, we employed BMGY medium (Buffered Glycerol complex Medium) and BMMY medium (Buffered Methanol complex Medium). For the preparative production, BMGH medium (Buffered Minimal Glycerol medium) and BMMH medium (Buffered Minimal Methanol medium) were used. Methanol (0.5 % *v*/*v*) was added every 24 h to the cultures for inducing the β-*N*-acetylhexosaminidase expression. On day 5 after the first inoculation, the cultures were purified as described previously [13] using cation exchange chromatography column (Fractogel SO_3_^−^) of GE Healthcare, Pittsburgh, PA, USA, equilibrated with 10 mM sodium citrate-phosphate buffer pH 3.5. The elution of the enzyme was done with a linear gradient of 0–1 M NaCl (60 mL, 2 mL/min). The concentration of protein was assayed according to Bradford [31] using Protein Assay Dye Reagent Concentrate (Bio-Rad, Watford, Hertfordshire, UK) calibrated for γ-globulin from bovine plasma (IgG, BioRad, Watford, Hertfordshire, UK). The yield of this one-step purification was approximately 70–85% (100% is the amount of crude enzyme produced in the cultivation medium). The enzyme purity was determined by SDS-PAGE using 10 % polyacrylamide gel. The purified enzymes were stored in McIlvaine buffer (50 mM citric acid/100 mM Na_2_HPO_4_) at pH 5.0.

### 4.3. Standard Assay for Enzyme Activity

The GlcNAcase of *Tf*Hex variants was measured in end-point assays using the substrate of *p*-nitrophenyl 2-acetamido-2-deoxy-β-d-glucopyranoside (*p*NP-GlcNAc; Sigma–Aldrich, USA) at a starting concentration of 2 mM. The reaction mixture was incubated in McIlvaine buffer (50 mM citric acid/100 mM Na_2_HPO_4_ pH 5.0) for 10 min at 35 °C and 850 rpm. Then, the reaction (50 µL) was stopped by mixing with 0.1 M Na_2_CO_3_ (1 mL). The concentration of released *p*-nitrophenol was determined at 420 nm. One unit of enzymatic activity corresponds to the amount of enzyme releasing 1 µmol of *p*-nitrophenol per minute under the above conditions. The GalNAcase activity was measured correspondingly, using *p*-nitrophenyl 2-acetamido-2-deoxy-β-d-galactopyranoside (**1**; *p*NP-GalNAc; Sigma–Aldrich, USA). All data were measured in triplicate.

The effect of pH on the activity of *Tf*Hex variants was measured at 35 °C in the same way as above. To cover the wide pH range of 2–10, Britton-Robinson buffer (0.04 M H_3_PO_4_, 0.04 M phenylacetic acid, 0.04 M H_3_BO_3_/0.2 M NaOH) was used. All data were measured in triplicate.

Michaelis–Menten parameters of hydrolysis of *p*NP-GlcNAc and *p*NP-GalNAc by *Tf*Hex variants were determined in a discontinuous kinetic assay (a continuous assay with *p*NP-substrates at pH 5 exhibits a very low sensitivity). The reaction mixtures (400 µL) containing substrates at a concentration of 0.01–5.0 mM in McIlvaine buffer pH 5.0 were incubated at 25 °C and 850 rpm for 10–15 min. Aliquots (50 µL) were taken regularly and the reactions were stopped by adding to 100 µL of 1 M Na_2_CO_3_. Absorbance (420 nm) was determined by the Spectra Max Plus spectrophotometer (Molecular Devices, Sunnyvale, CA, USA). All data were measured in duplicate. The initial reaction velocities were determined by linear regression of respective time points. The kinetic constants (*K*_m_, *k*_cat_) were extracted using GraphPad Prism 7 (GraphPad Software, La Jolla, CA, USA).

### 4.4. Molecular Modeling, Docking and Molecular Dynamics

For modeling of *Tf*Hex mutant variants and molecular dynamics simulations we used the homology model of *Tf*Hex described previously [22]. Models of mutant variants were shortly minimized in vacuo with NOVA force field in YASARA, using positional restraints for residues that were far from mutated residues. Models of substrates were built by YASARA [32] and docked by GLIDE from Schrödinger software [33] in minimized structures. 

Substrate-enzyme complexes were solvated with TIP3 water model with density of 0.997 g/mL, the protonation state of amino acid residues was modeled at pH 6, the protonation of selected active site amino acid residues was corrected manually. Complexes were neutralized by sodium ions and optimized by short minimization with YASARA. Parameters used for molecular dynamics simulation and minimization were similar: periodic boundary condition, YASARA2 force field, Paricle-Mesh Ewald method for long range interactions, NPT (constant number of particles/pressure/temperature) ensemble with Berendsen thermostat and barostat, and AutoSMILES algorithm [34] were used for ligand parameterization. 

Molecular dynamics simulations were analyzed with YASARA macros. Selected representative complexes were minimized with OPLS 5 force filed by Protein Preparation Wizard from Schrodinger software and used for GLIDE docking. Binding scores for substrates in the active site of enzymes were calculated by rigid docking with XP scoring method, found to be a superior method for substrate ranging [35], and averaged. The GLIDE XP score is calculated as follows:

XP GLIDE Score = *E*_coul_ + *E*_vdW_ + *E*_bind_ + *E*_penalty_ (1)

*E*_bind_ = *E*_hyd_enclosure_ + *E*_hb_nn_motif_ + *E*_hh_cc_motif_ + *E*_PI_ + *E*_hb_pair_ + *E*_phobic_pair_ (2)

*E*_penalty_ = *E*_desolv_ + *E*_ligand_strain_ (3)

It includes an electrostatic term (*E*_coul_) and a term for van der Waals interactions (*E*_vdW_). *E*_bind_ includes contributions of hydrophobic interactions and hydrogen bonds to the binding, input of *π*-stacking and *π*-cation interaction. The penalty term of binding includes an estimate of strain energy (unfavorable contacts), and desolvation of the substrate or protein. 

### 4.5. Compound (t-Butoxycarbonylamino)ethylthioureidyl 2-acetamido-2-deoxy-β-d-glucopyranoside (***2***)

The title compound **2** was prepared from GlcNAc based on the procedure described previously [36], see also Supplementary Materials (Scheme S1). The ^1^H and ^13^C NMR data were consistent with the structure (Supplementary Materials, Table S1, Figures S1 and S4).

### 4.6. Compound (t-Butoxycarbonylamino)ethylthioureidyl 2-acetamido-2-deoxy-β-d-galactopyranosyl-(1→4)-2-acetamido-2-deoxy-β-d-glucopyranoside (***3***)

*p*NP-GalNAc (**1**; 82 mg, 0.24 mmol), acceptor **2** (304 mg, 0.72 mmol), and Tyr470Phe *Tf*Hex (3.6 U, 15.6 mg, 270 μL) were incubated in a mixture of acetonitrile (40 % *v*/*v*) and McIlvaine buffer pH 5.0 (total reaction volume 4.8 mL) at 35 °C and 850 rpm. The reaction was monitored by HPLC and TLC (propan-2-ol/water/NH_4_OH aq., 7:2:1). After 2.25 h, another batch of donor **1** (82 mg, 0.24 mmol) was added. After 6.25 h, the reaction was stopped by boiling for 2 min, and upon cooling down to room temperature, the reaction mixture was centrifuged (13,500 rpm; 10 min) and loaded onto a Biogel P-2 column (2.6 × 100 cm, Bio-Rad, Watford, Hertfordshire, UK) using water as a mobile phase at an elution rate of 7.3 mL/h. Pure acceptor **2** was partially recovered (141 mg). The fractions containing the product were combined and lyophilized; the title compound **3** was obtained as a white solid (123 mg, 0.2 mmol, 51 % isolated yield). HRMS (ESI^+^): found *m*/*z* 648.25211 (expected 648.25212 for [M + Na]^+^, C_24_H_43_N_5_O_12_SNa). For the respective NMR and MS data, see the Supplementary Materials (Table S2, Figures S2 and S5). Prior to conjugation to a multivalent carrier, the *t*-butoxycarbonyl protecting group is deprotected by dissolving compound **3** (20 mM) in 1M HCl and incubating under stirring at 4 °C for 48 h. The reaction mixture is neutralized by adding anex resin in OH^−^ cycle and lyophilized. The deprotected amine **4** (Supplementary Materials, Table S3, Figures S3 and S6) is rather prone to decomposition under long-term storage.

## 5. Conclusions

The present study demonstrates a successful combination of protein and reaction engineering in the large-scale synthesis of a bioactive disaccharide GalNAcβ4GlcNAc, carrying an amino function for multivalent conjugation. This disaccharide is a selective ligand of galectin-3 with a potential application in biomedicine. We have demonstrated the astonishing stabilizing effect of the Tyr470Phe mutation on GalNAcase activity of *Tf*Hex under various conditions, namely over a large span of pH and in the presence of water-miscible co-solvents. Thus, apart from a considerable increase in transglycosylation capability and suppression of the undesired side hydrolysis, this point mutation brings a significant stabilization of GalNAcase activity under harsh conditions and the resulting catalyst is highly suitable as a versatile tool for preparative GalNAc-ylations.

## Figures and Tables

**Table 1:** Purification yields of *Tf*Hex mutants and GalNAcase/GlcNAcase activity ratios.

Enzyme	Protein Yield [mg] ^1^	GlcNAcase [U/mg]	GalNAcase [U/mg]	GalNAcase/GlcNAcase Ratio
WT *Tf*Hex	188	37.4	45.1	1.2
Tyr470Phe *Tf*Hex	308	0.3	0.3	1.0
Tyr470His *Tf*Hex	84	0.4	0.1	0.3
Tyr470Asn *Tf*Hex	225	1.6	0.2	0.1

Note: ^1^ Purification from 800 mL cultivation medium.

**Table 2:** Kinetic parameters of *p*NP-GlcNAc and *p*NP-GalNAc hydrolysis by *Tf*Hex variants.

Enzyme	Substrate	*K*_m_ [mM]	*k*_cat_ [s^−1^]	*k_cat_*/*K_m_* [s^−1^ mM^−1^]
WT TfHex	*p*NP-GlcNAc	0.11 ± 0.02	47 ± 1	434
	*p*NP-GalNAc	0.69 ± 0.09	104 ± 5	150
Tyr470Phe TfHex	*p*NP-GlcNAc	0.023 ± 0.008	0.27 ± 0.01	12
	*p*NP-GalNAc	0.050 ± 0.005	0.166 ± 0.003	3.3
Tyr470His TfHex	*p*NP-GlcNAc	0.053 ± 0.008	0.67 ± 0.02	13
	*p*NP-GalNAc	0.35 ± 0.03	0.188 ± 0.004	0.53
Tyr470Asn TfHex	*p*NP-GlcNAc	0.89 ± 0.13	11.6 ± 0.6	13
	*p*NP-GalNAc	0.89 ± 0.09	0.35 ± 0.01	0.40

**Table 3:** Binding XP scores and hydrogen bonding of docked substrates and hydrolytic products. ^a^

*Tf*Hex variant	Binding XP scores [kJ/mol] (*averaged number of hydrogen bonds*)			
	*p*NP-GlcNAc	*p*NP-GalNAc	GlcNAc	GalNAc
WT	−38.5 (*6.0*)	−34.9 (*5.5*)	−23.5	−29.5
Tyr470Phe	−33.2 (*4.7*)	−36.5 (*5.0*)	−32.0	−23.4
Tyr70His (positive)	−32.2 (*5.6*)	−25.7 (*3.3*)	−27.1	−29.4
Tyr470His (neutral)	−37.0 (*4.6*)	−32.0 (*4.6*)	−22.6	−31.5
Tyr470Asn	−31.1 (*3.8*)	−30.8 (*4.7*)	−27.6	−23.5

Note: ^a^ A lower score corresponds to a more favorable binding when a compound creates more hydrogen bonding, hydrophobic, and electrostatic interactions with active site residues.

## Supplementary Materials

The following are available online at http://www.mdpi.com/1420-3049/24/3/599/s1: Synthesis of acceptor 2 (Scheme S1), NMR analysis of acceptor **2** and product **3** (Tables S1–S3; Figures S1–S3), MS analysis of compounds **2**, **3, 4** (Figures S4–S6), SDS-PAGE of *Tf*Hex WT, Tyr470Phe, Tyr470His and Tyr470Asn *Tf*Hex (Figure S7), molecular modeling, and molecular dynamics simulations (Figures S8–S11).
