# Supplementary material for: The β-N-Acetylhexosaminidase in the Synthesis of Bioactive Glycans: Protein and Reaction Engineering
Source: Molecules. 2019 Feb 8;24(3):599. doi: 10.3390/molecules24030599 (PMC6384963; doi:10.3390/molecules24030599)
Supplement: Supplementary file 1 [file molecules-24-00599-s001.pdf]

molecules

Supplementary Materials

The β-*N*-Acetylhexosaminidase in the Synthesis of Bioactive Glycans: Protein and Reaction Engineering

Pavla Bojarová[1,*], Natalia Kulik[2], Michaela Hovorková[1], Kristýna Slámová[1] , Helena Pelantová[3] and Vladimír Křen[1]

1 Laboratory of Biotransformation, Institute of Microbiology, Czech Academy of Sciences, Vídeňská 1083, CZ-14220 Praha 4, Czech Republic; bojarova@biomed.cas.cz (P. B.); michaela.hovorkova@natur.cuni.cz (M. H.); slamova.kristyna@gmail.com (K. S.); kren@biomed.cas.cz (V. K.)
2 Center for Nanobiology and Structural Biology, Institute of Microbiology, Czech Academy of Sciences, Zámek 136, CZ-37333 Nové Hrady, Czech Republic; kulik@nh.cas.cz (N. K.)
3 Laboratory of Molecular Structure Characterization, Institute of Microbiology, Czech Academy of Sciences, Vídeňská 1083, CZ-14220 Praha 4, Czech Republic; pelantova@biomed.cas.cz (H. P.)
* Correspondence: bojarova@biomed.cas.cz; Tel.: +420-296-442-360

Received: date; Accepted: date; Published: date

Abstract: *N*-Acetylhexosamine oligosaccharides terminated with GalNAc act as selective ligands of galectin-3, a biomedically important human lectin. Their synthesis can be accomplished by β-*N*-acetylhexosaminidases (EC 3.2.1.52). Advantageously, these enzymes tolerate the presence of functional groups in the substrate molecule, such as the thiourea linker useful for covalent conjugation of glycans to a multivalent carrier, affording glyconjugates. β-*N*-Acetylhexosaminidases exhibit activity towards both *N*-acetylglucosamine (GlcNAc) and *N*-acetylgalactosamine (GalNAc) moieties. A point mutation of active-site amino acid Tyr to other amino acid residues, especially Phe, His and Asn, has previously been shown to strongly suppress the hydrolytic activity of β-*N*-acetylhexosaminidases, creating enzymatic synthetic engines. In the present work, we demonstrate that Tyr470 is an important mutation hotspot for altering the ratio of GlcNAcase/GalNAcase activity, resulting in mutant enzymes with varying affinity to GlcNAc/GalNAc substrates. The enzyme selectivity may additionally be manipulated by altering the reaction medium upon changing pH or adding selected organic co-solvents. As a result, we are able to fine-tune the β-*N*-acetylhexosaminidase affinity and selectivity, resulting in a high-yield production of the functionalized GalNAcβ4GlcNAc disaccharide, a selective ligand of galectin-3.

Contents

1. Synthesis of acceptor **2** (Scheme S1)
2. ^{1}H, ^{13}C NMR analysis of acceptor **2,** products **3** and **4** (Tables S1, S2, S3; Figures S1, S2, S3)
3. MS analysis of compounds **2**, **3**, **4** (Figures S4, S5, S6)
4. SDS-PAGE of *Tf*Hex WT, Tyr470Phe, Tyr470His and Tyr470Asn *Tf*Hex (Figure S7)
5. Molecular modeling and molecular dynamics simulations (Figures S8-S11)

1. Synthesis of acceptor 2

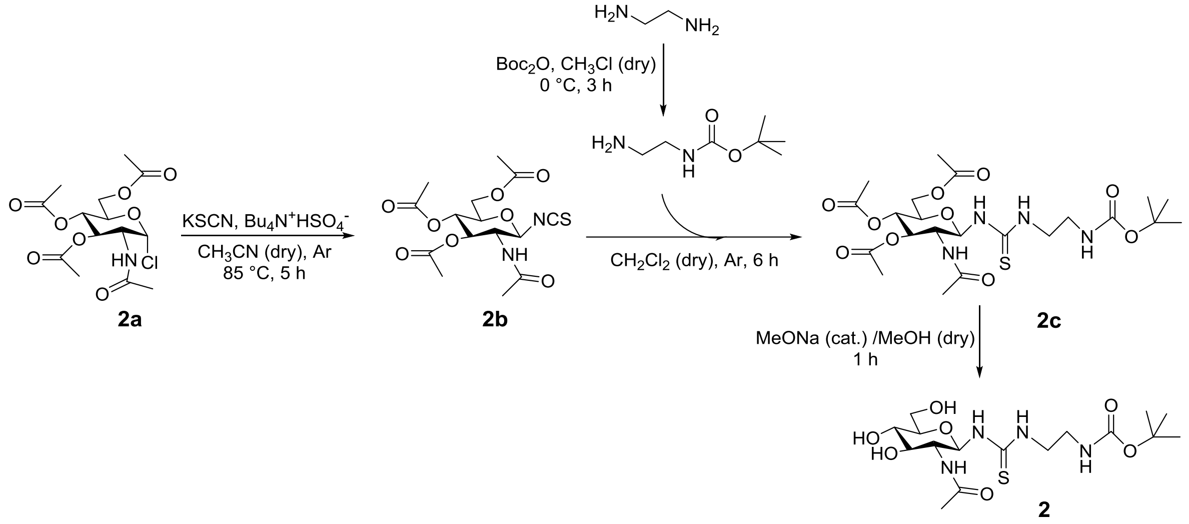

Scheme S1. Synthesis of acceptor **2** by adopted procedure based on Sauerzapfe et al. [1].

In the first step, 2-acetamido-3,4,6-tri-*O*-acetyl-2-deoxy-β-D-glucopyranosyl chloride **2a** (7 g, 19.1 mmol) [2], KSCN (3.73 g, 38.2 mmol), and tetrabutylammonium hydrogen sulfate (6.52 g, 19.1 mmol) were dissolved in dry acetonitrile (210 mL) with molecular sieve 4Å (10 g) and left stirring under argon at 85 °C for 5h. Then, it was left overnight at room temperature. The reaction mixture was filtered, evaporated *in vacuo* to dryness and purified by column chromatography (EtOAc/PE, 2/1) to afford 2-acetamido-3,4,6-tri-*O*-acetyl-2-deoxy-β-D-glucopyranosyl isothiocyanate **2b** (3 g, 7.7 mmol; 41%).

As the next step, ethylenediamine (6.11 g, 102 mmol) was dissolved in dry chloroform (50 mL) under argon and immersed to ice-cold bath. A solution of Boc-anhydride (2.28 g, 10.1 mmol) in dry chloroform (50 mL) was added dropwise. Reaction mixture was kept in an ice-cold bath for 3 h, then it was left to proceed at r.t. for 48h. The reaction was quenched by the addition of distilled water (50 mL). Organic phase was washed with brine (3×40 mL), water phase was washed with chloroform (4×20 mL), organic phases were combined, dried over Na_2SO_4 and evaporated to yield *tert*-butyl (2-aminoethyl)carbamate as brownish oil (1.51 g, 9.4 mmol; 93%). Prior to the conjugation step, this compound was thoroughly dried under vacuum.

In the last conjugation step, **2b** (1.55 g, 4.0 mmol) was combined with thoroughly dried *tert*-butyl (2-aminoethyl)carbamate under argon and dissolved in dry CH_2Cl_2 (40 mL). The reaction proceeded for 6h, then it was quenched by adding distilled water (40 mL). Organic phase was washed with water (3×10 mL) while water phase was washed with CH_2Cl_2 (2×20 mL). Combined organics were washed with brine, dried over Na_2SO_4, then evaporated and purified by column chromatography (EtOAc/PE, 6/4) to obtain peracetylated product **2c** (2.17 g, 4.0 mmol; 99%). This compound was subsequently reacted under Zemplén conditions (catalytic amount of NaOMe, dry MeOH) to yield the desired deacetylated acceptor **2** (1.7 g, 99%).

2. NMR analysis of acceptor 2 and product 3

Table S1. ^{1}H and ^{13}C NMR data of acceptor **2** (399.87 MHz for ^{1}H, 100.55 MHz for ^{13}C, D_2O, 30 °C).

	Atom	δ_C	m.	δ_H	n_H	m.	*J*[Hz]
**Boc**	**CO**	158.47	S	-	0		
	**C**	81.37	S	-	0		
	**(CH$_3$)$_3$**	27.96	Q	1.218	9	s	
**spacer**	**1′**	44.37	T	3.39[a]	2	m	
	**2′**	39.44	T	3.046	2	m	

	**CS**	n.d.	S	-	0		
**GlcNAc**	**1**	83.26	D	5.258	1	br s	
	**2**	54.81	D	3.665	1	m	
	**3**	74.36	D	3.423	1	dd	10.2, 8.6
	**4**	69.94	D	3.265	1	dd	9.9, 8.6
	**5**	77.52	D	3.325	1	ddd	9.9, 5.0, 2.1
	**6**	60.88	T	3.683	1	dd	12.4, 2.1
				3.546	1	dd	12.4, 5.0
	**2-CO**	175.27	S	-	0		
	**Ac**	22.31	Q	1.805	3	s	

n.d. ... not detected; [a] HSQC readout

a

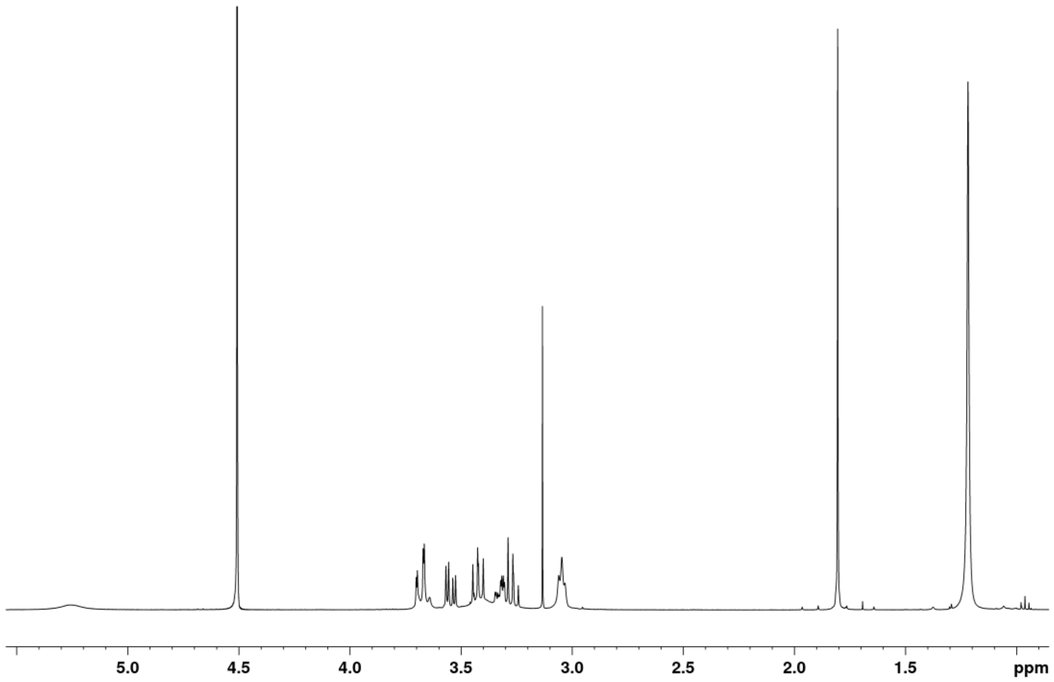

b

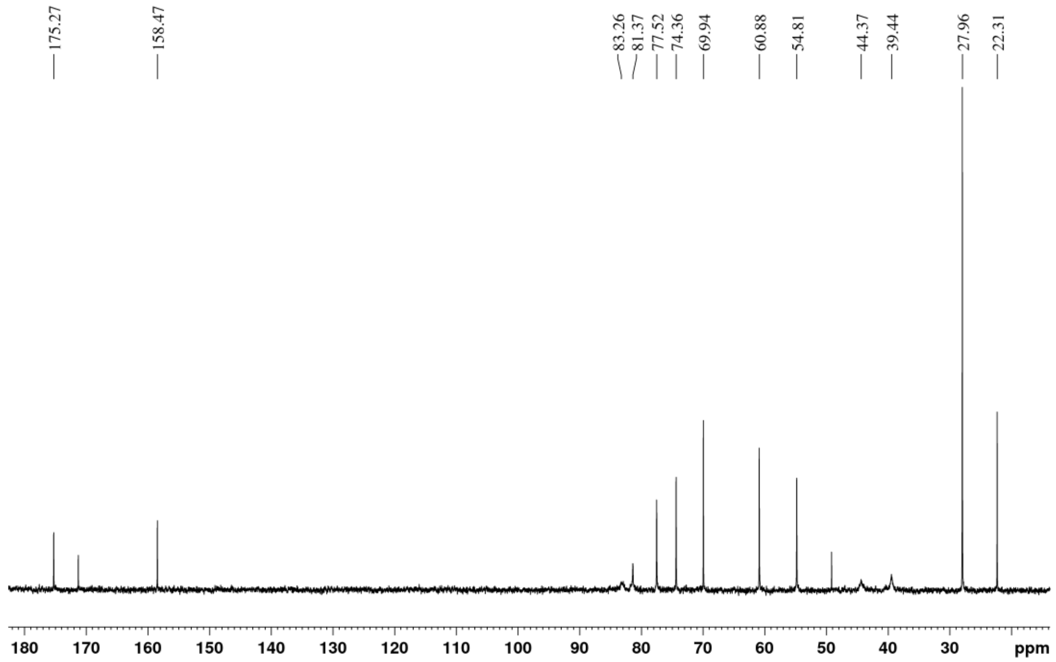

Figure S1. (a) ^{1}H and (b) ^{13}C NMR spectra of acceptor **2** (399.87 MHz for ^{1}H, 100.55 MHz for ^{13}C, D_2O, 30 °C).

Table S2. ^{1}H and ^{13}C NMR data of disaccharide **3** (700.13 MHz for ^{1}H, 176.05 MHz for ^{13}C, D_2O, 30 °C)

	**Atom**	δ_C	**m.**	δ_H	**n**$_H$	**m.**	***J*** **[Hz]**
**Boc**	**CO**	158.49	S	-	0		
	**C**	81.34	S	-	0		
	**(CH$_3$)$_3$**	27.96	Q	1.217	9	s	
**spacer**	**1′**	44.61	T	3.45[a]	2	m	
	**2′**	39.56	T	3.050	2	m	
	**CS**	n.d.	S	-	0		
**GlcNAc**	**1**	82.87	D	5.365,	1	br s	
				5.240			
	**2**	54.08	D	3.687	1	m	
	**3**	72.96	D	3.60[a]	1	m	
	**4**	78.92	D	3.458	1	m	
	**5**	76.05	D	3.395	1	m	
	**6**	60.24	T	3.638	1	m	
				3.442	1	m	
	**2-CO**	175.31	S	-	0		
	**Ac**	22.29	Q	1.802	3	s	
**GalNAc**	**1**	101.94	D	4.328	1	d	8.4
	**2**	52.84	D	3.725	1	dd	10.8, 8.4

	**3**	70.94	D	3.546	1	dd	10.8, 3.4
	**4**	67.90	D	3.735	1	d	3.4
	**5**	75.60	D	3.518	1	dd	8.2, 4.0
	**6**	61.23	T	3.596	1	dd	11.7, 8.2
				3.562	1	dd	11.7, 4.0
	**2-CO**	175.02	S		0		
	**Ac**	22.46	Q	1.855	3	s	

n.d. ... not detected; [a] HSQC readout

a

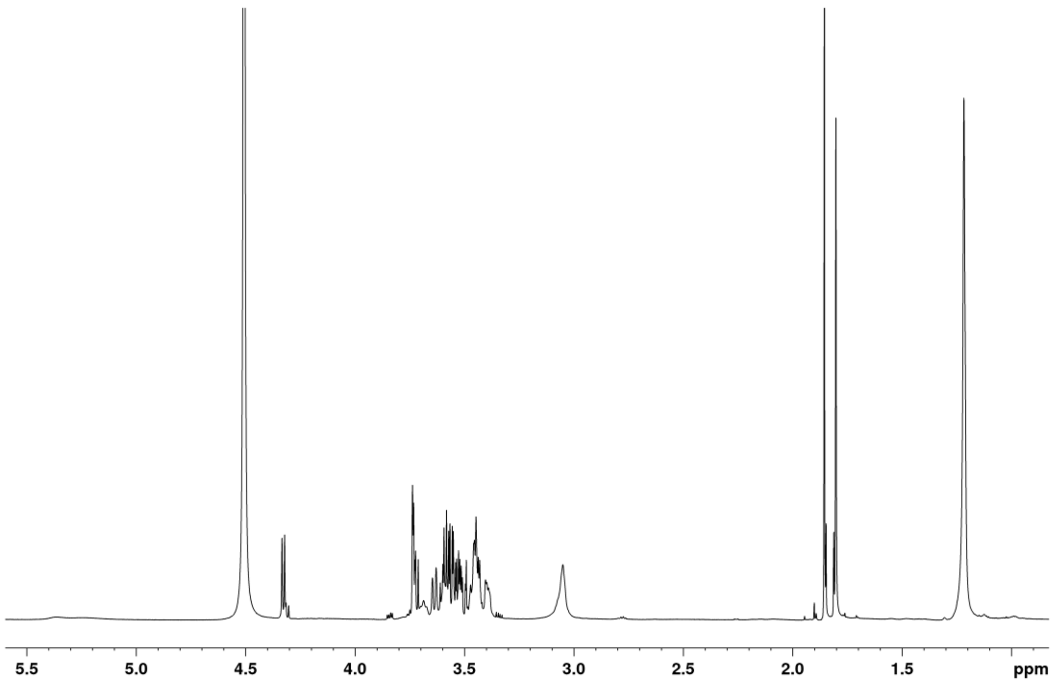

b

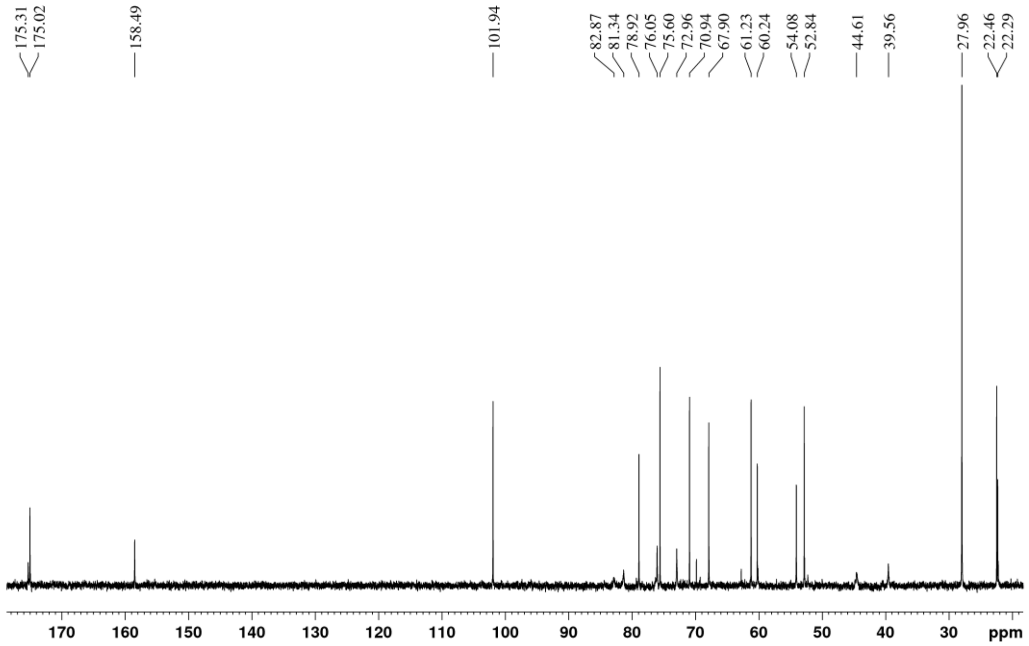

Figure S2. (a) ^{1}H and (b) ^{13}C NMR spectra of disaccharide **3** (700.13 MHz for ^{1}H, 176.05 MHz for ^{13}C, D_2O, 30 °C).

Table S3. ^{1}H and ^{13}C NMR data of disaccharide **4** (399.87 MHz for ^{1}H, 100.55 MHz for ^{13}C, D_2O, 30 °C)

	**Atom**	δ_C	**m.**	δ_H	n_H	**m.**	***J* [Hz]**
**spacer**	**1′**	42.91	T	3.66[a]	1	m	
				3.55[a]	1	m	
	**2′**	39.39	T	2.91[a]	2	m	
	**CS**	n.d.	S	-	0		
**GlcNAc**	**1**	83.0[a]	D	5.313	1	br s	
	**2**	54.00	D	3.71[a]	1	m	
	**3**	72.97	D	3.60[a]	1	m	
	**4**	78.93	D	3.46[a]	1	m	
	**5**	76.17	D	3.41[a]	1	m	
	**6**	60.24	T	3.63[a]	1	m	
				3.44[a]	1	m	
	**2-CO**	175.29	S	-	0		
	**Ac**	22.26	Q	1.793	3	s	
**GalNAc**	**1**	101.94	D	4.327	1	d	8.4
	**2**	52.84	D	3.72[a]	1	m	
	**3**	70.91	D	3.55[a]	1	m	
	**4**	67.89	D	3.73[a]	1	m	
	**5**	75.61	D	3.52[a]	1	m	
	**6**	61.24	T	3.58[a]	2	m	
	**2-CO**	175.04	S	-	0	m	

	**Ac**	22.46	Q	1.852	3	s	

n.d. ... not detected; [a] HSQC readout

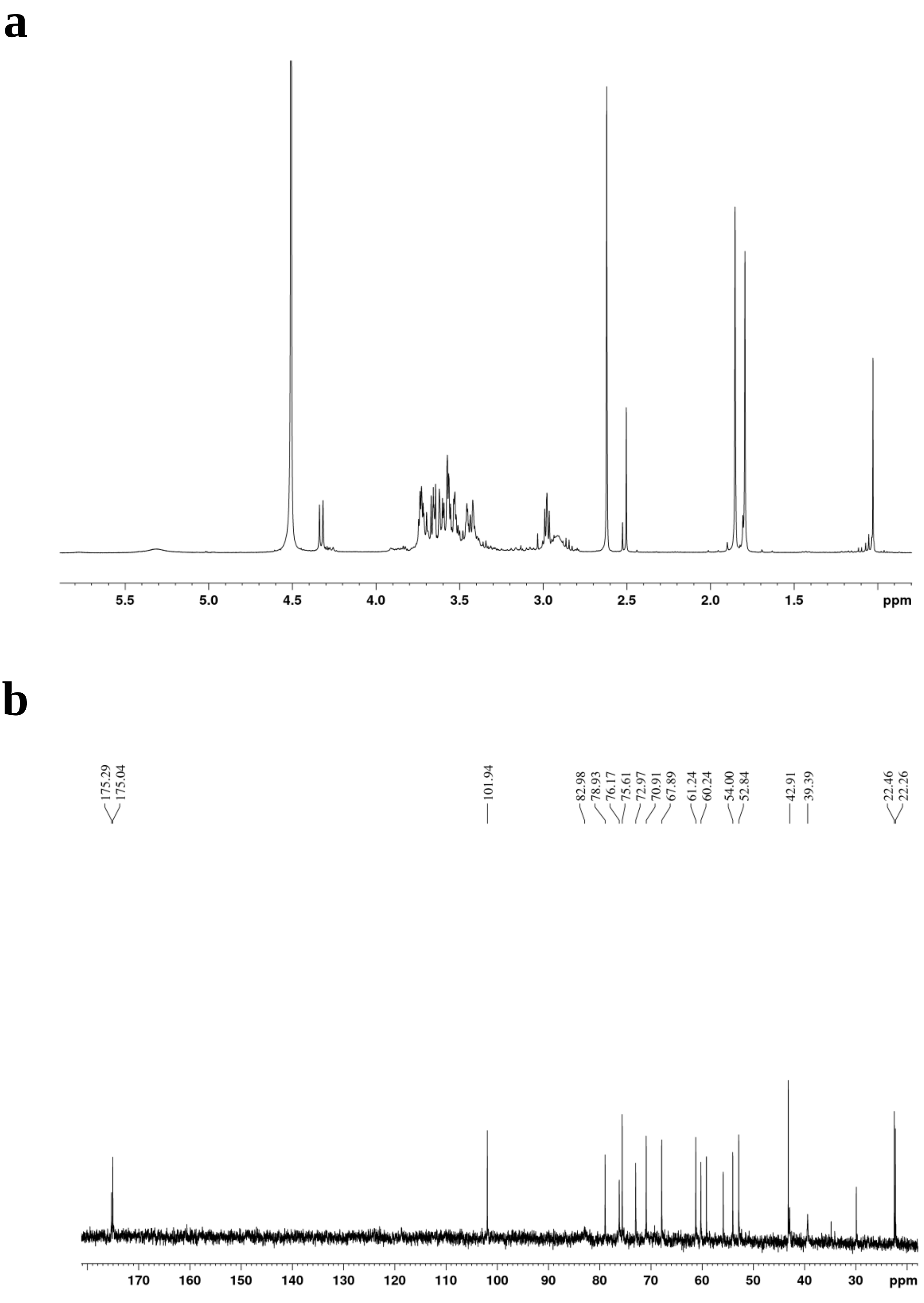

Figure S3. (a) ^{1}H and (b) ^{13}C NMR spectra of disaccharide **4** (399.87 MHz for ^{1}H, 100.55 MHz for ^{13}C, D_2O, 30 °C).

3. MS analysis of acceptor 2, products 3 and 4

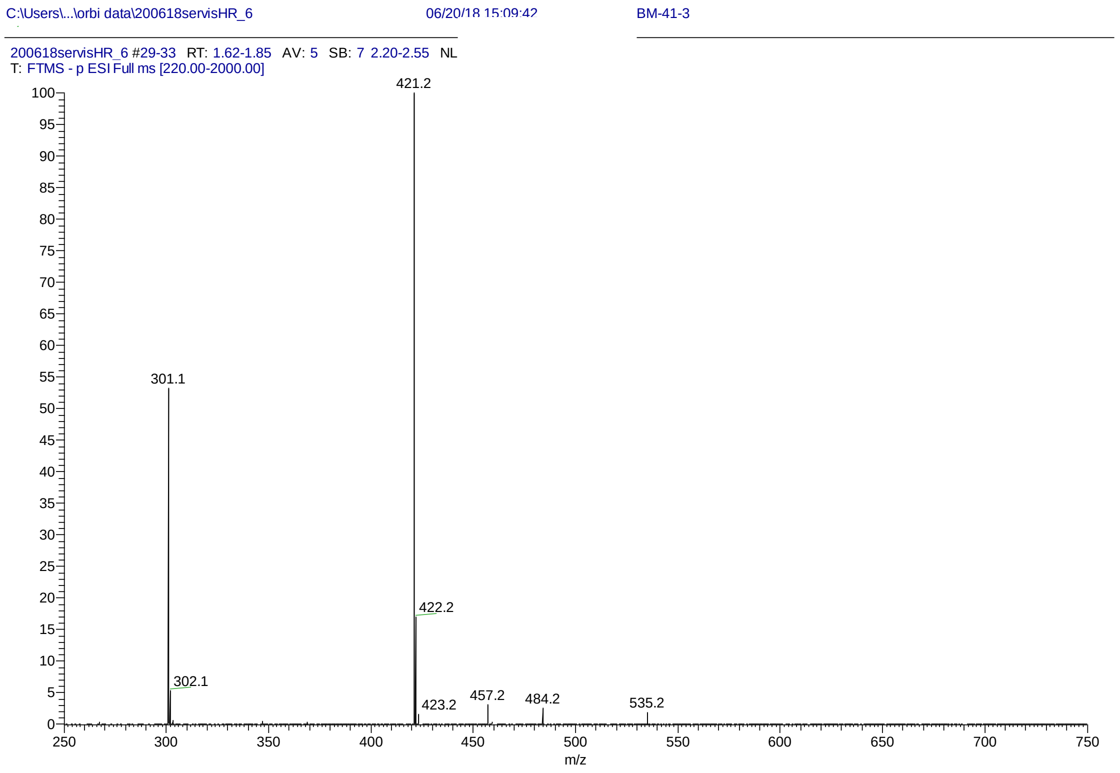

Figure S4. MS spectrum (ESI⁻) of product **3** ($[M - H]^-$, *m/z* 421.2; $[M + Cl]^-$, *m/z* 457.2; $[M + NO_3]^-$, *m/z* 484.2).

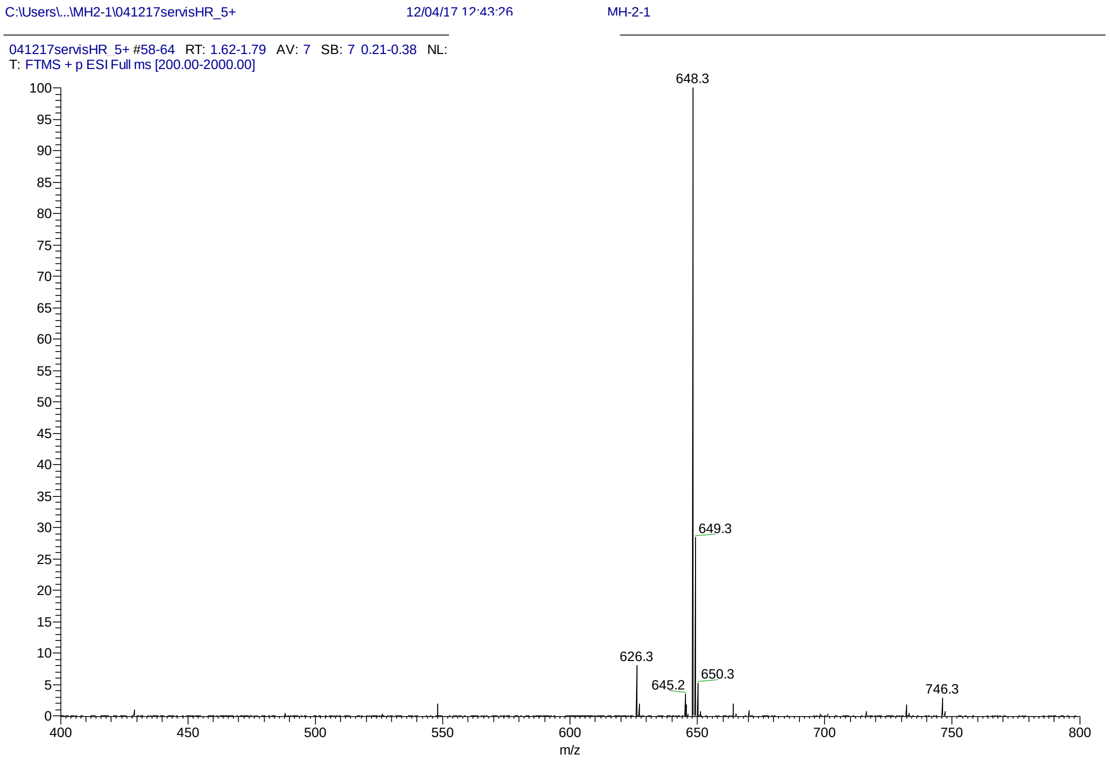

Figure S5. MS spectrum (ESI+) of product **3** ($[M + Na]^+$, *m/z* 648.3; $[M + H]^+$, *m/z* 626.3).

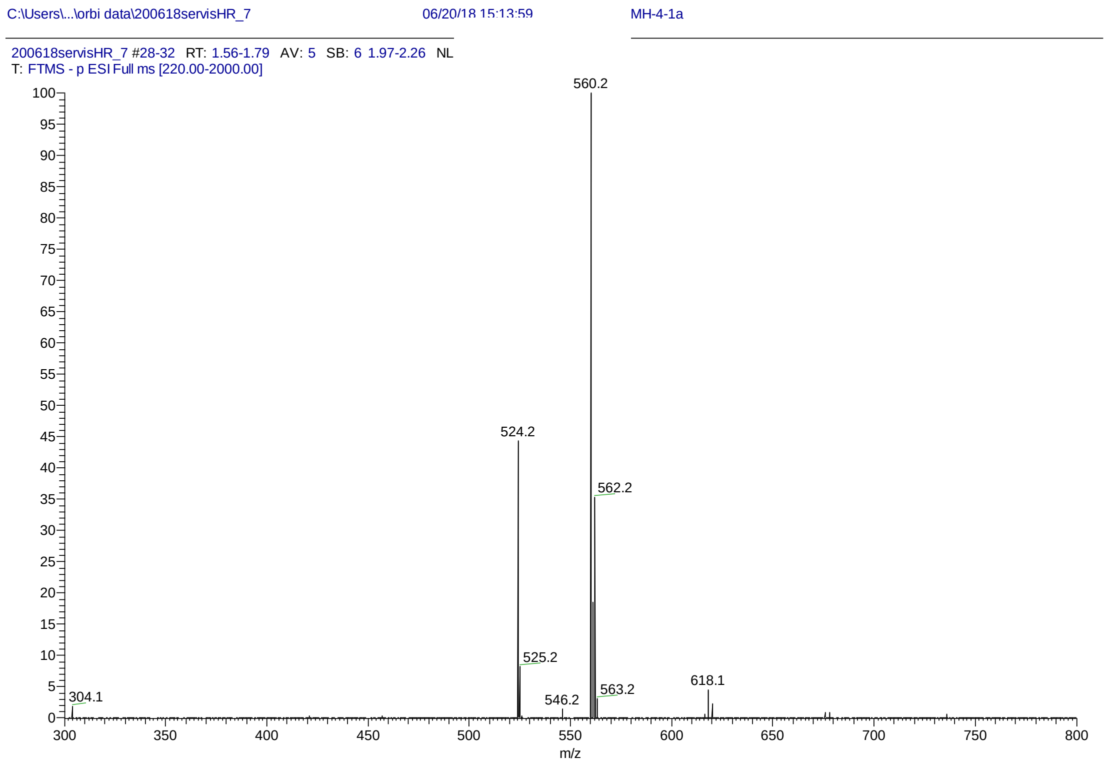

Figure S6. MS spectrum (ESI$^-$) of compound **4** ([M - H]$^-$, *m/z* 524.2; [M - 2H + Na]$^-$, *m/z* 546.2; [M + Cl]$^-$, *m/z* 560.2). HRMS (ESI$^-$): calculated (for $C_{19}H_{34}O_{10}N_5S^-$) 524.20319, measured 524.20245 (-1.40 ppm).

4. SDS-PAGE of *Tf*Hex WT, Tyr470Phe, Tyr470His and Tyr470Asn *Tf*Hex

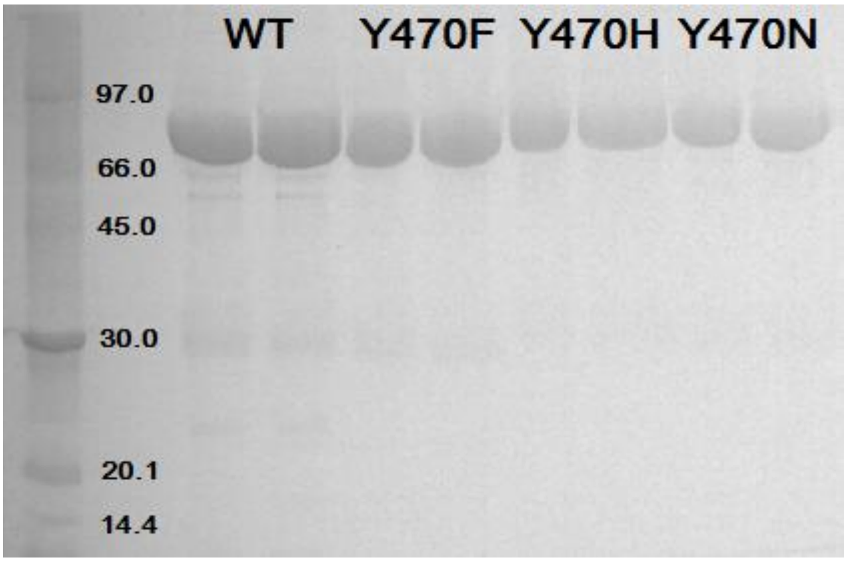

Figure S7. SDS-PAGE of mutant variants of *Tf*Hex WT, Tyr470Phe, Tyr470His and Tyr470Asn *Tf*Hex.

5. Molecular modeling and molecular dynamics simulations

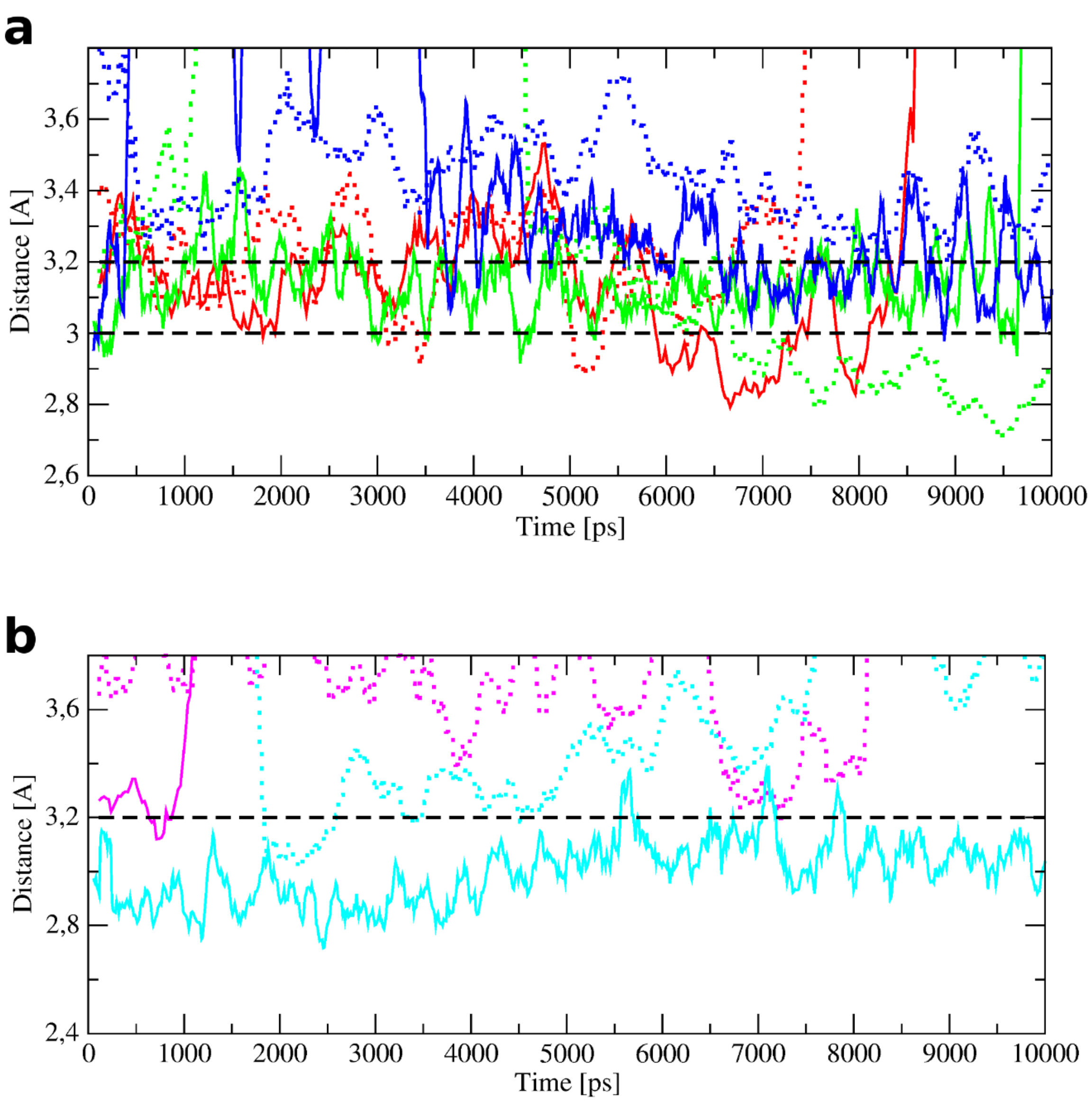

Figure S8. Distance between the carboxylic oxygen of catalytic Glu371 and the glycosidic oxygen of *p*NP-GlcNAc and *p*NP-GalNAc substrates in the active site of *Tf*Hex variants during molecular dynamic simulation. (**a**), The simulation of substrates docked into the WT (**red**), Tyr470Phe (**green**), and Tyr470His (**blue**) variants; (**b**), the simulation of substrates docked into the Tyr470His (neutral) variant in **magenta**, Tyr470His (positive) variant in **turquoise**. The simulation with *p*NP-GlcNAc is depicted in a full line, with *p*NP-GalNAc in a dotted line. The distance within 2.5-3.2 Å corresponds to weak hydrogen bond interaction and above 3.2 Å to a weak electrostatic [3]. The graphs are prepared with XMGRACE and data are averaged over 10 steps [4].

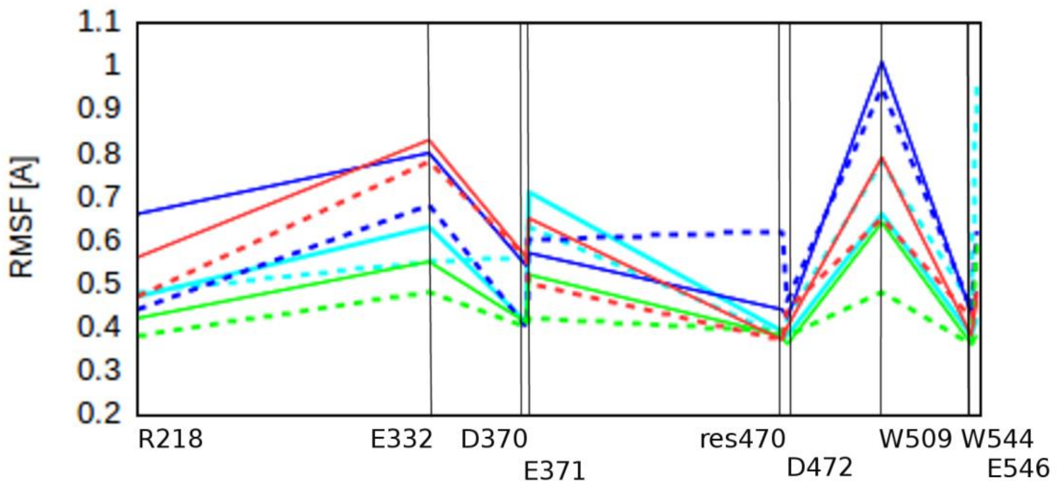

Figure S9. RMSF of active site residues in *Tf*Hex variants during a stable period of molecular dynamics run (2-10 ns). WT is shown in **red**, Tyr470Phe in **green**, Tyr470His (positive) in **blue**, Tyr470Asn in **turquoise**. The simulation with *p*NP-GlcNAc is depicted in a full line, with *p*NP-GalNAc in a dotted line. Res470 is Tyr, Phe, His or Asn, depending on the particular variant.

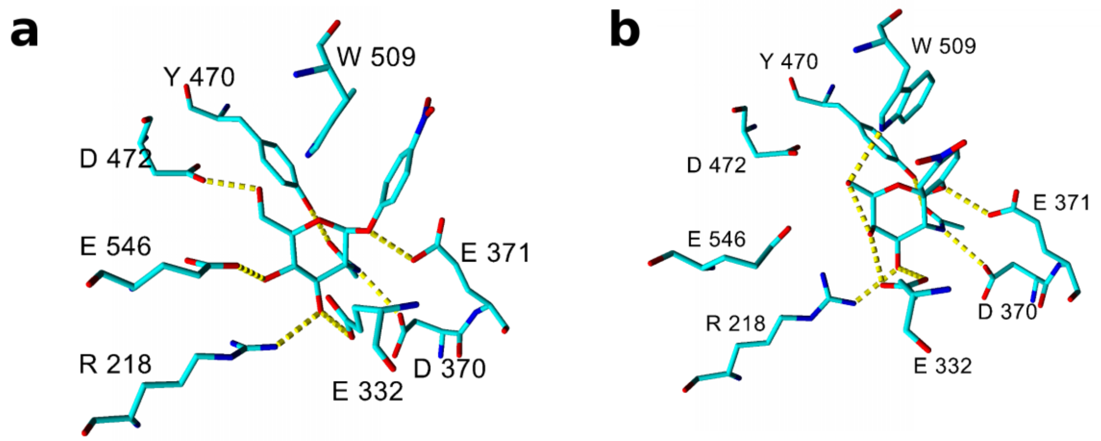

Figure S10. Complex of *p*NP-GlcNAc (a) and *p*NP-GalNAc (b) in the active site of *Tf*Hex WT after a stable period of molecular dynamics simulation.

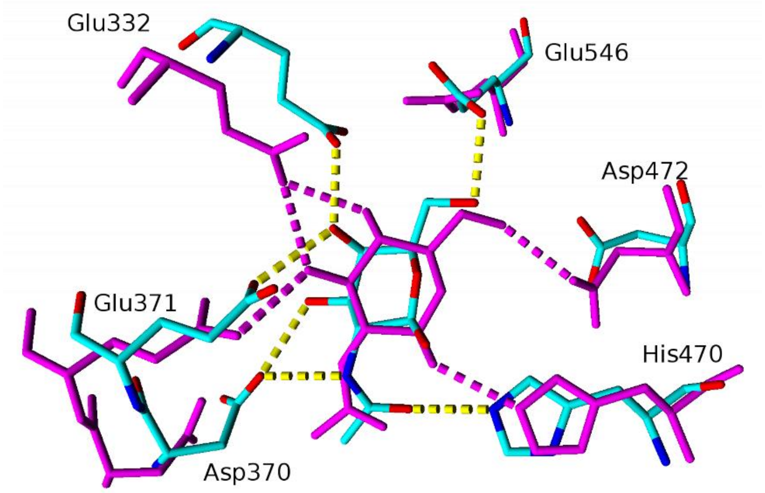

Figure S11. Overlay of complexes of hydrolytic products with the Tyr470His (positive) mutant variant after 10 ns of molecular dynamics simulation. GalNAc-Tyr470His complex is in element colors, GlcNAc-Tyr470His complex is in **magenta**. Hydrogen bonds are shown by dashed line. The position of both hydrolytic product is completely different from the respective *p*NP-substrates. As a result, the residue Arg218 does not interact with either product and the distance between the carbohydrate oxygen at C-1 and the oxygen of catalytic Glu371 is too far for a nucleophilic attack (4.7 and 5.6 Å for GalNAc and GlcNAc, respectively).

References

1. Sauerzapfe, B.; Křenek, K.; Schmiedel, J.; Wakarchuk, W.W.; Pelantová, H.; Křen, V.; Elling, L. Chemo-enzymatic synthesis of poly-*N*-acetyllactosamine (poly-LacNAc) structures and their characterization for CGL2-galectin-mediated binding of ECM glycoproteins to biomaterial surfaces. *Glycoconj. J.* **2009**, *26*, 141–159, doi:101007/s10719-008-9172-2.
2. Fialová, P.; Weignerová, L.; Rauvolfová, J.; Přikrylová, V.; Pišvejcová, A.; Ettrich, R.; Kuzma, M.; Sedmera, P.; Křen, V. Hydrolytic and transglycosylation reactions of *N*-acyl modified substrates catalysed by β-*N*-acetylhexosaminidases. *Tetrahedron* **2004**, *60*, 693-701, doi: https://doi.org/10.1016/j.tet.2003.10.111.
3. Jeffrey, G. A. *An introduction to hydrogen bonding*; Oxford University Press: Oxford, UK, 1997; ISBN 0-19-509549-9.
4. Turner, P.J. *XMGRACE, Version 5.1.19*; Center for Coastal and Land-Margin Research, Oregon Graduate Institute of Science and Technology: Beaverton, OR, USA, 2005.
